# Supplementary material for: Impacts of a record-breaking storm on physical and biogeochemical regimes along a catchment-to-coast continuum
Source: PLoS One. 2020 Jul 28;15(7):e0235963. doi: 10.1371/journal.pone.0235963 (PMC7386647; doi:10.1371/journal.pone.0235963)
Supplement: Attachment — Submitted filename: Response to Reviewers.docx [file pone.0235963.s002.docx]

We sincerely thank the reviewer for taking the time to evaluate our manuscript and providing positive feedback in relation to the study. We have incorporated changes to the manuscript and here provide a response to each of the reviewer’s comments.

5. Review Comments to the Author

This study has provided a unique angel to investigate the impact of storms on a catchment-to-coast continuum, and presented the results of an original research with a large amount of solid data. Experiments design, and specifically how each parameter was calculated, was described in sufficient details. The conclusions drawn in this work was clear and was well supported by the data. I generally agree the publication of this work, as long as the authors can take care of several minor comments listed here:

Thank you very much for the encouraging feedback and your views on the overall quality of the study

The title of this work is the impact of a storm on physical and biogeochemical regimes. This manuscript has provided very solid results on the physical part, but only briefly mentioned biogeochemistry throughout the work (e.g., pH, DO). In the discussion session, the authors did talk about the impact of the storm on the lake system and the downstream coastal system, but the discussions were more like speculation without support of actual data. The manuscript will be strengthened if the authors could include some data about how the nutrients regime changed in the lead up to and immediate aftermath of storm Desmond in both the lake and the downstream coastal regions.

We agree that the primary results of the study did focus more on the physical aspects, although this was largely related to the physical impacts being much more profound compared with the biogeochemical changes primarily due to the timing of the storm (winter when productivity is low). We had envisaged that this in and of itself is a useful finding.

We do include some analysis of pH and dissolved oxygen as the reviewer notes and did show how pH dropped in the freshwater lake immediately post-storm and also the implications of large river flows for reinforcing stratified conditions that promote deoxygenation in the estuarine zone. We also showed how turbidity increased in the aftermath of the storm (Fig. 3B ; line 359) and a portion of the results and discussion highlight the reduction in salinity in the Lough Furnace estuarine basin (e.g. lines 474-481) and overall freshening effect of such a storm on coastal systems. For example the section on storm impacts on estuarine salt balance in the discussion (beginning lines 616)

Chlorophyll *a* is monitored on both systems but did not show a significant change given the winter setting. We reference how the timing of this storm was important in minimising some biogeochemical changes and compare it directly to the large chlorophyll *a* changes observed during a summer storm in the same system (reference 17 and Lines 514-517).

Finally, nitrogen, phosphorus and water colour are analysed from water samples taken on both Feeagh and Furnace but only at a monthly frequency so regrettably we were unable to definitively assess how the nutrient status changed in the immediate lead-up to and aftermath of the storm. However, we do note that these monthly values did not differ from historical values (Line 521-522), again showing that for the freshwater lake at least biogeochemical changes were small compared to big chemical changes in salt and oxygen in the estuary.

Some specific minor comments are:
Line 96: There is a typo. It should be “between” not “0between”.

Corrected

Line 473: Should be “(C) salt flux” not “(B) salt flux”.

Corrected

--------------------------------------------------------------------------------------------------------------------------

Additional journal requirements

1. Please ensure that your manuscript meets PLOS ONE's style requirements, including those for file naming.

We have edited aspects of the manuscript style format to meet the requirements. We have also re-uploaded each of the files using the correct naming format (e.g. Fig 1.eps is now Fig1.eps)

2. We note that Figure 1 in your submission contains a map image which may be copyrighted.

We require you to either (a) present written permission from the copyright holder to publish these figures specifically under the CC BY 4.0 license, or (b) remove the figures from your submission:

We have supplied a replacement Figure 1. The basemap background map layer in the original figure, which contained the copyrighted material, has been removed for the replacement figure.

Updated request for revised map source material on 12/05/2020:

1. Thank you for updating the basemap of Fig. 1. To ensure that this figure is now compliant with CC BY 4.0 (<https://creativecommons.org/licenses/by/4.0/>), we ask that you provide us with source information for the basemap, the data points, and the data overlaid on the map.

In the revised Fig. 1 the following data sources where used:

1. Lake shapefiles – from Environmental Protection Agency and available here: <http://gis.epa.ie/geonetwork/srv/eng/catalog.search#/metadata/c4040e19-38ec-4120-a588-8cd01ac94a9c>. Data is for public use under Creative Commons CC-By 4.0.
2. River shapefiles – from Environmental Protection Agency and available here: <http://gis.epa.ie/geonetwork/srv/eng/catalog.search#/metadata/c4043e19-38ec-4120-a588-8cd01ac94a9c>. Data is for public use under Creative Commons CC-By 4.0.
3. Data points (coloured, labelled markers on the map) – these are the location of instruments deployed by the authors of this study and have been marked according to GPS coordinates recorded during each deployment.
4. The original basemap, which had the copyrighted material, has been removed for the revised Fig. 1. **The current revised Fig. 1 does not contain any basemap** and we thus we cannot provide any source information.
5. We have updated the caption of Fig.1 to provide the general source information for the lake and river shapefiles listed in point 1 (<http://gis.epa.ie/GetData/Download>).
